# *Cladosporium cladosporioides* from the perspectives of medical and biotechnological approaches

**DOI:** 10.1007/s13205-015-0323-4

**Published:** 2015-12-31

**Authors:** Manaf AlMatar, Essam A. Makky

**Affiliations:** 1 Department of Biotechnology, Institute of Natural and Applied Sciences (Fen Bilimleri Enstitüsü), Cukurova University, Rectorate 01330 Balcali, Adana, Turkey; 2 Department of Biotechnology, Faculty of Industrial Sciences and Technology, Universiti Malaysia Pahang (UMP), 26300 Gambang, Kuantan, Malaysia

**Keywords:** Calphostin (C), Chlorpyrifos, *Cladosporium cladosporioides*, Ergosterol peroxide (EP), p-Methylbenzoic acid, Pectin methylesterase (PME), Polygalacturonase (PG)

## Abstract

Fungi are important natural product sources that have enormous potential for the production of novel compounds for use in pharmacology, agricultural applications and industry. Compared with other natural sources such as plants, fungi are highly diverse but understudied. However, research on *Cladosporium cladosporioides* revealed the existence of bioactive products such as p-methylbenzoic acid, ergosterol peroxide (EP) and calphostin C as well as enzymes including pectin methylesterase (PME), polygalacturonase (PG) and chlorpyrifos hydrolase. p-Methylbenzoic acid has ability to synthesise 1,5-benzodiazepine and its derivatives, polyethylene terephthalate and eicosapentaenoic acid. EP has anticancer, antiangiogenic, antibacterial, anti-oxidative and immunosuppressive properties. Calphostin C inhibits protein kinase C (PKC) by inactivating both PKC-epsilon and PKC-alpha. In addition, calphostin C stimulates apoptosis in WEHI-231 cells and vascular smooth muscle cells. Based on the stimulation of endoplasmic reticulum stress in some types of cancer, calphostin C has also been evaluated as a potential photodynamic therapeutic agent. Methylesterase (PME) and PG have garnered attention because of their usage in the food processing industry and significant physiological function in plants. Chlorpyrifos, a human, animal and plant toxin, can be degraded and eliminated by chlorpyrifos hydrolase.

## Introduction

The genus *Cladosporium*, identified by Link for the first time in 1815, is characterised by the absence of a sexual proliferation phase; therefore, it is classified into the Fungi Imperfecti (Deuteromycota) group. This genus belongs to the mitosporic Ascomycotic phylum, subphylum Pezizomycota, class Dothideomycetes, family Mycosphaerellaceae and contains approximately 500 species (De Hoog et al. 1995; Okada et al. 1996). *Cladosporium*, species are most frequently found in outdoor and indoor environments, spoiled organic matter and are considered as food important contaminants (Dixon and Polak-Wyss 1991; De Hoog et al. 2000; San-Martin et al. 2005). Additionally, some *Cladosporium* spp. can develop even on the surface of glass fibres and inside water pipes (Macher 1999; Johanning 1999). These fungi can utilise different growth substrates, such as, wood plants, dead plants, food, soil, straw and textiles (Tasić and Miladinović-Tasić 2007). Several species of this genus have been associated with fish diseases (Otto 2000). The common ancestor has been identified for only 15 species of *Cladosporium*. The most isolated species are *Cladosporium sphaerospermum*, *Cladosporium cladosporioides*, *Cladosporium herbarum* and *Cladosporium elatum* (De Hoog et al. 1995; Masclaux et al. 1995). In contrast, many species of *Cladosporium* are also able to produce some secondary metabolites such as, antibiotics which are inhibitors of *Bacillus subtilis*, *Escherichia coli* and *Candida albicans* (Gallo et al. 2004). Furthermore, some *Cladosporium* species are efficient biological insecticides, particularly against insects that have developed resistance to chemical insecticides (Abdel-Baky and Abdel-Salam 2003).

*Cladosporium cladosporioides* normally grows on potato-dextrose agar (PDA) medium and generates one-celled conidia (spores). It is recognised by the formation of 3-cm diameter colonies, which are either (the colonies are either) olive-green or brown basal side (Krogh 1989; Tasić and Miladinović-Tasić 2007). In this review, we critically summarise the medical and industrial usage of bioactive compounds and enzymes derived from *C. cladosporioides*.

## Bioactive metabolites from *Cladosporium cladosporioides*

### p-Methylbenzoic acid

**Fig. 1:**
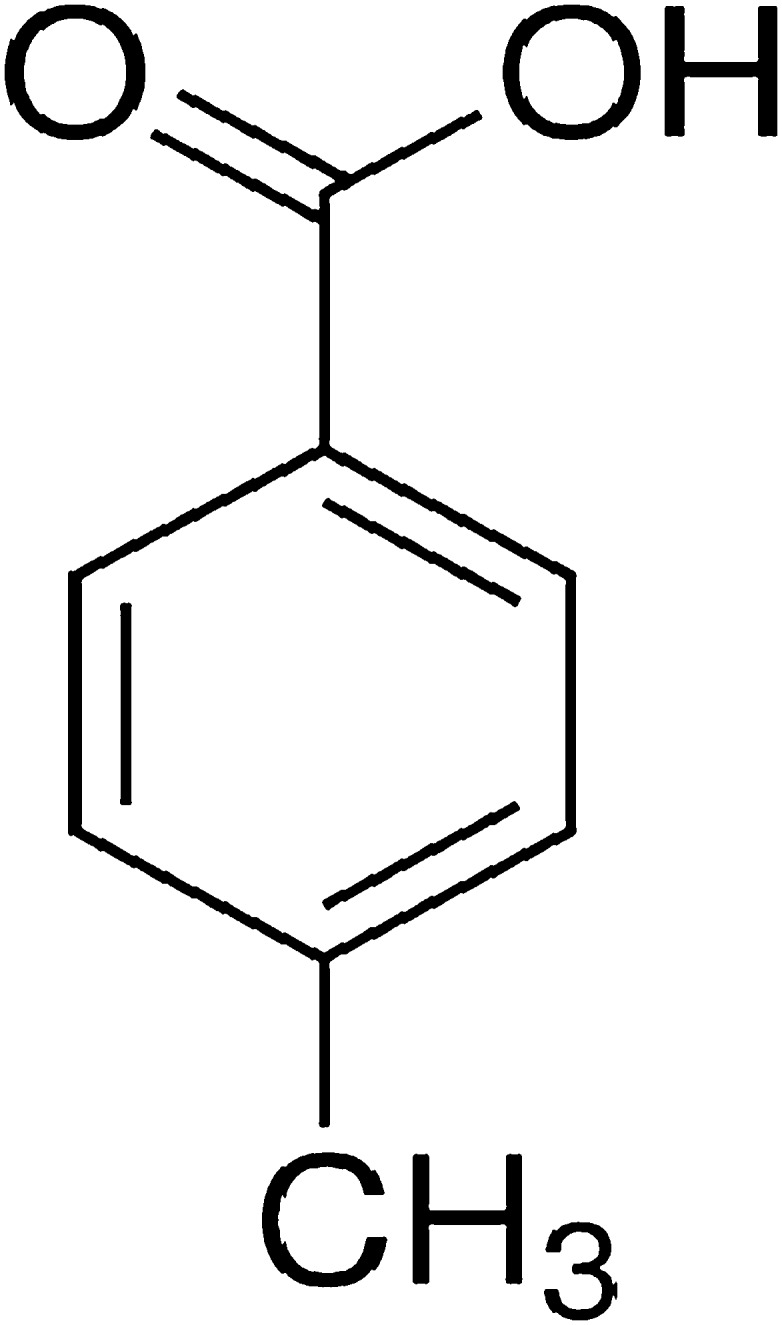
p-Toluic acid structure

p-Methylbenzoic acid (Fig. 1), known as p-toluic acid, was extracted from *C. cladosporioides* an isolate from marine sponge and later identified using spectroscopic methods (San-Martin et al. 2005). p-Methylbenzoic acid promotes the synthesis of 1,5-benzodiazepine and its derivatives, which are active compounds against diverse targets such as interleukin converting enzymes (ICE), potassium blockers (I_k_) and peptide hormones (CCK) (Herpin et al. 2000; Varala et al. 2007). Benzodiazepines are widely used as antianxiety, anticonvulsant, anti-depressive, anti-inflammatory agents, analgesics and sedatives (Tsoleridis et al. 2008). Benzodiazepines have a variety of activities including antiulcer, antileukaemic, vasopressin antagonist, antiplatelet and endothelial antagonist and relief of skeletal muscle joint pain (Kumar and Joshi 2007; Aasth et al. 2013). Benzodiazepines are also used as fibre dyes and are used to produce fused ring compounds such as oxadiazolo-, triazolo-, oxazino- or furano benzodiazepines (Wu et al. 2006; Aasth et al. 2013). Initially used to yield polyethylene terephthalate (PET) via condensation polymerisation with ethylene glycol, p-methylbenzoic acid is an intermediate in the production of terephthalic acid (Speight 1999). PET is widely used commercially and has one of the highest manufactured tonnages of all polymer products in the world (Jankauskaitė et al. 2008). PET is used to produce fabrics, fibres and textiles. In addition, PET has progressively dominated the bottle market for bottled water and carbonated soft drinks as well as food containers and packaging materials. These days, PET is increasingly being used to produce fibre strengthened composite (Giles and Bain 2001; Vakili and Fard 2010).

Based on studies, Shirasaka et al. (2005) demonstrated that p-methylbenzoic affect fatty acid composition by increasing eicosapentaenoic acid (EPA) production. EPA, a (n-3) long-chain polyunsaturated fatty acid (PUFA), serves as the phospholipid acyl group in membrane-bound organisms ranging from bacteria to humans. EPA contributes to membrane organisation, cell division and resistance to oxidative stress at low temperatures in psychrophiles and marine bacteria (Nishida et al. 2006; Okuyama et al. 2007; Kawamoto et al. 2009).

Consuming EPA, one of the primary v-3 fatty acids in fish oils, has been demonstrated to be beneficial in several experimental and clinical studies. A standard dose of EPA inhibits tumour growth-induced lipolysis and muscle protein degradation, possibly by suppressing the cytokine IL-6 and reducing a tumour-specific product that is a proteolysis-inducing factor (PIF) EPA improved prolonged survival, enhanced weight maintenance, ameliorated food intake and reduced protein degradation without effecting protein synthesis. These observations indicate that EPA may be useful for the clinical management of malignancy patients (Beck et al. 1991; Babcock et al. 2000a). The American Heart Association (AHA) recommends that patients suffering from heart diseases such as coronary heart disease should supplement their diet with (1 g) EPA and docosahexaenoic acid (DHA) daily. The AHA also recommends that healthy adults consume one serving of fish at least two times per week (Manhiani et al. 2009). Anti-inflammatory activity has specifically been demonstrated for EPA, but not for all v-3 fatty acids. However, regioisomeric mixtures of EPA and epoxydocosapentaenoic acid (EDP) appear to be highly efficient for reducing inflammatory pain (Beck et al. 1991; Inceoglu et al. 2011). Doses of 1.25–2.5 g/kg body weight have been observed to produce the optimal effect when using EPA (Babcock et al. 2000b).

### Ergosterol peroxide (EP)

**Fig. 2:**
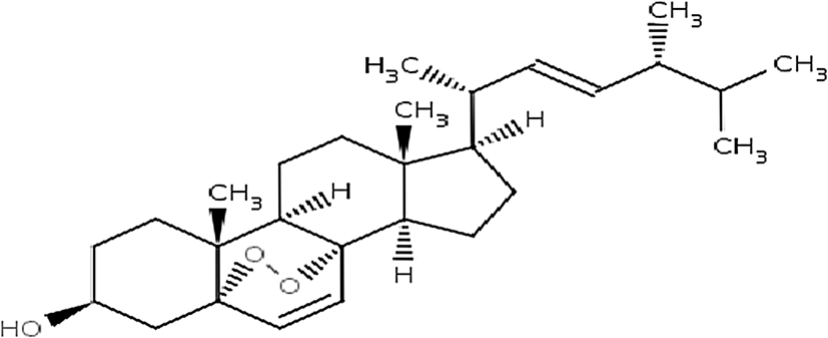
Ergosterol peroxide (EP) structure

Ergosterol peroxide (EP) (Fig. 2), a bioactive compound, has been extracted from *C. cladosporioides* obtained from a marine sponge using spectroscopic methods (San-Martin et al. 2005). EP isolated from *C. cladosporioides* has not been examined against various types of diseases. However, EP isolated from different organisms ranging from fungi to plants has been reported to have anticancer, antiangiogenic, antibacterial, anti-oxidative and immunosuppressive properties (Rhee et al. 2012). EP, known as pro-vitamin D_2_, contributes to the prevention of colon and prostate cancer (Kobori et al. 2007). EP inhibits phorbol-12-myristate 13-acetate (TPA), TPA-induced inflammation and tumour promotion in mice (Kobori et al. 2007). EP also inhibits the growth of certain cancer cells, stimulates apoptosis in HL60 human leukaemia cells, reduces lipid peroxidation in rat liver microsomes and represses the production of mitogen stimulated mouse and human lymphocytes (Yasukawa et al. 1994; Fujimoto et al. 1994; Bok et al. 1999; Yaoita et al. 2002; Guyton et al. 2003; Kuo et al. 2003; Takei et al. 2005; Prompiboon et al. 2008).

According to Rhee et al. (2012), nontoxic concentrations of EP suppress the DNA-binding activity, phosphorylation, nuclear trans-localisation of signal transducer and activator of transcription 3 (STAT3) in U266 cells. EP inhibits the phosphorylation of the upstream kinase, Janus kinase 2 (JAK2) and other tyrosine kinase family members, i.e., Src homology region 2 domain-containing phosphatase-1 (SHP-1). SHP-1 protein and mRNA levels are increased by EP; conversely, silencing the SHP-1 gene abrogates EP-mediated STAT3 inhibition (Rhee et al. 2012). Furthermore, cellular and protein levels of vascular endothelial growth factor (VEGF), a STAT3 target gene, are decreased by EP. Female BALB/c athymic nude mice inoculated with U266 cells have significantly reduced tumour growth when treated with EP (Rhee et al. 2012). Additionally, EP treatment reduced STAT3 and CD34 expression in tumours (Rhee et al. 2012). It has been demonstrated that EP suppresses LPS-induced inflammatory responses by inhibiting NF-kB and C/EBPb transcriptional activity and phosphorylation of MAP kinases (MAPKs) (Kobori et al. 2007). EP further suppresses cell growth and STAT1-mediated inflammatory responses by altering the redox status in HT29 cells (Kobori et al. 2007).

EP is a highly efficient antimicrobial agent against the Gram-positive pyogenic bacterium, *Staphylococcus aureus*, with an MIC value of 50 mg/ml (Huong et al. 2010; San-Martin et al. 2005). EP also exhibits cytotoxic activity against RD cancer cells with an IC_50_ of 4.6 mg/ml (Huong et al. 2010). Recent studies indicate oncogene and tumour suppressor gene expression is controlled by RNAs referred to as microRNAs (miRNAs) (Calin and Croce 2006; Lee et al. 2007). The expression of certain microRNAs (miRNAs) is increased in cancer and drug-resistant cells (Zheng et al. 2010). EP effectively stimulated death in miR-378 cells at low concentrations. Therefore, EP may be a promising novel therapeutic agent against drug-resistant tumour cell (Wu et al. 2012).

### Calphostin (C)

**Fig. 3:**
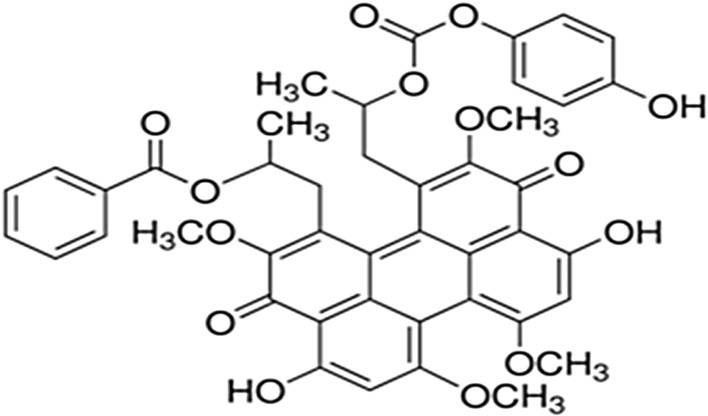
Calphostin (C) structure

Calphostin C (Fig. 3), isolated from *C. cladosporioides* culture broth, inhibits protein kinase C (PKC) by inactivating both PKC-epsilon [a Ca(2+)-independent novel isoform] and PKC-alpha [a Ca(2+)-dependent conventional isoform] and exhibits cytotoxic activity against various tumour cells (Rotenberg et al. 1995). A 500 nM calphostin C concentration was used to test a series of N-terminal-truncated bovine PKC-alpha mutants expressed in *Saccharomyces*. This concentration was active against proteins with up to 91 amino acid (aa) deletions from the amino terminus (ND91), while only 20 % inhibition occurred against a mutant protein truncated by 140 aa (ND140). Therefore, the amino acid (aa) sequence from 92 to 140 contains the structural element of PKC-alpha that is suppressed by calphostin C (Kobayashi et al. 1989; Rotenberg et al. 1995). Calphostin C reduces cancer cell lung colonisation and adherence to the endothelium for both high and low metastatic sub-populations at sub-micromolar IC_50_ concentrations (Liu et al. 1992). At a 3 µM concentration, calphostin C repressed ISO+ ionomycin-induced cAMP and cGMP accretion by 80 and 78 %, respectively (Ogiwara et al. 1998). The effects of calphostin C in vitro have been assessed using ɛ-peptide, ζ-peptide and Ac-MBP (4–14) as substrates (Ogiwara et al. 1998). The percent inhibition of Ac-MBP (4–14) phosphorylation was 58 % at a 1 µM calphostin C concentration. However, calphostin C reduced phosphorylation by 60 and 50 % when ɛ-peptide and ζ-peptide were used as substrates, respectively (Ogiwara et al. 1998).

WEHI-231 cells undergo apoptosis when calphostin C is used at a 250 nM concentration (Chmura et al. 1996). The addition of calphostin C led to increased ceramide production compared with baseline levels and a simultaneous decrease in sphingomyelin; therefore, calphostin C synergistically acted with exogenous ceramide analogues to induce apoptosis (Chmura et al. 1996). These findings imply that the relationship between PKC activity and ceramide is antagonistic to the signalling events prior to apoptosis (Chmura et al. 1996). Potent inhibitors of phospholipase (PLDs) have an anti-apoptotic effect in vascular smooth muscle cells (VSMCs); however, PLD activity is suppressed by calphostin C, resulting in VSMC apoptosis. Calphostin C also increases microtubule disturbances, suggesting that calphostin C may be used to destabilise microtubule networks. Consequently, calphostin C induces VSMC apoptosis by inhibiting PLD activity followed by microtubule polymerisation (Zheng et al. 2004). The earliest calphostin C effect is the destruction of glycoprotein export from the endoplasmic reticulum (ER), accompanied by ER-originated vacuole creation. Vacuolisation of the ER is associated with ER stress response induction, which includes the activation of c-Jun N-terminal kinase, protein kinase R-like ER kinase and increased expression of CCAAT/enhancer binding protein homologous transcription factor (CHOP; GADD153). These effects indicate that ER stress is the result of calphostin C interaction with targets other than PKC (Kaul and Maltese 2009). Cell sensitivity to calphostin C decreased when CHOP expression was reduced, suggesting that calphostin C apoptosis induction via ER stress is due to the interruption of ER morphology and transport function. Because calphostin C can elicit ER stress, it has been evaluated as a potential photodynamic therapy agent against certain cancers (Kaul and Maltese 2009).

## Enzymes secreted by *Cladosporium cladosporioides*

### Pectin methylesterase (PME) and polygalacturonase (PG)

The pectinase enzymes PME and PG have been reported to be excreted by *C. cladosporioides* and were extracted using the Buescher and Furmanski procedure (Bastos et al. 2013). The ideal conditions to produce both PME and PG are after 10 days of incubation and precipitation with (NH_4_)_2_SO_4_ and benzoate buffer at pH 4.0 (Buescher and Furmanski 1978; Silva et al. 2008; Bastos et al. 2013). Pectinases, or pectinolytic enzymes, are found in plants, bacteria, insects and fungi and can be categorised into three main groups: pectinesterases, protopectinases and depolymerases. Pectin de-esterification catalysed by esterase enzymes leads to the removal of the metoxyl ester group. The degradation of insoluble protopectin is performed by protopectinases, resulting in highly polymerised soluble pectin. However, hydrolysis of the α (1 → 4) glycosidic linkage in pectic acid is catalysed by depolymerases (Whitaker et al. 2003; Jayani et al. 2005; Gonzalez and Rosso 2011). PME (EC 3.1.1.11) is responsible for pectin degradation by catalysing the demethoxylation of the homogalacturonan chain of pectin to release methanol and acidic pectin (Micheli 2001). Pectin is a primary component of plant cell walls; therefore, PME can affect the integrity and rigidity of plant tissues. PG catalyses the hydrolytic cleavage of the polygalacturonic acid chain via the introduction of water across the oxygen bridge, releasing galacturonic acid as the main product (Kluskens et al. 2005).

Recently, pectinases have garnered attention due to their application in the food processing industry and critical role in plant physiology (Dixit et al. 2013). The main industrial purposes of pectinases are to extract and clarify fruit and vegetable juice (Alkorta et al. 1998). Pectin is responsible for the consistency and turbidity of juice, resulting in increased viscosity that prevents juice concentration, clarification and filtration. Pectin degradation is achieved by adding pectolytic enzymes leading to high juice yields by allowing additional clarification and filtering as well as reduced viscosity (Sarιoğlu et al. 2001; De Gregorio et al. 2002; Souza et al. 2003; Fernández-González et al. 2004; Ribeiro et al. 2010). Unicellular products, formed by the transformation of organised tissues into a suspension of intact cells, can be produced by pectinase enzymes (Kashyap et al. 2001). These products can be used in pulpy juices and nectars, baby foods, dairy products such as yogurt and puddings and protoplasts for diverse biotechnological applications (Kashyap et al. 2001). Vegetable oil extraction has been performed using pectinase enzymes. Oil yield and stability are improved after enzyme treatment and polyphenol and vitamin E content are increased, improving the oil’s organoleptic quality (Kashyap et al. 2001; Hoondal et al. 2002; Iconomou et al. 2010). Pectolytic microorganisms are also used to ferment coffee beans, a process that removes the mucilage layer from the beans (Avallone et al. 2002). For example, a commercial enzyme product containing pectinase is applied to coffee beans to initiate fermentation (Pasha et al. 2013). Significantly, fermentation time has been reduced by using pectinase enzymes (Amorim and Amorim 1977; Silva et al. 2000; Kashyap et al. 2001; Serrat et al. 2002). Chocolate flavour is developed by cocoa fermentation, which is performed using pectinolytic enzymes (Schwan and Wheals 2004; Ouattara et al. 2010). Tea leaf fermentation has been accelerated and facilitated by treatment with pectic enzymes at a dose adjusted to avoid leaf damage (Carr 1985; Kashyap et al. 2001). Pectolytic enzymes which have fungal origin facilitate the wine fermentation process and improve the quality and diversification of products (Sieiro et al. 2012). During the winemaking extraction process, pectic enzymes facilitate filtration and intensify wine flavour and colour (Sieiro et al. 2012).

### Chlorpyrifos hydrolase

Because of its use as a broad spectrum insecticide, chlorpyrifos (*O*,*O*-diethyl *O*-(3,5,6-trichloro-2-pyridyl) phosphorothioate) has been widely restricted in some European countries and the United states as it is potentially toxic and persistent in the environment (Li et al. 2007; Bhagobaty et al. 2006). 3,5,6-Trichloro-2-pyridinol (TCP) is a metabolite released from chlorpyrifos and chlorpyrifos-methyl during degradation (Xu et al. 2008; Anwar et al. 2009; Li et al. 2010). These compounds can be transported through the atmosphere and water and may transfer into soils and sediments, resulting in diffuse pollution (Harms et al. 2011). Oliver et al. (1999) demonstrated that chlorpyrifos is harmful to the respiratory system, central nervous system, cardiovascular system and reproductive system because of its highly potent toxicity. Their study was conducted in the United States at Columbia University in 263 pregnant females who were exposed to approximately to the same level of contamination. An acute relationship was observed between prenatal exposure to chlorpyrifos and low birth weight and smaller infant head size. Smaller head size is correlated with poor function and lower intelligence quotients (IQ). Chlorpyrifos’ toxic effects extend to diverse types of arthropods including ladybird beetles, bees and parasitic wasps (Lee et al. 2012). Chlorpyrifos also affects birds and fish, leading to reduced nestling weight and morphologic distortions for birds as well as death for both organisms (NCAP 2000). In plants, prolonged chlorpyrifos exposure leads to delayed seedling emergence, abnormal cell division and fruit malformation (Bhagobaty et al. 2006). Moreover, public attention to potential human health risks, from exposure to chlorpyrifos residue on food, has increased due to the frequent use of chlorpyrifos in agriculture (Cochran et al. 1995; Yu et al. 2006). Consequently, it is necessary to develop methods to degrade and eliminate chlorpyrifos from the environment. Although several bacterial isolates have demonstrated the ability to degrade chlorpyrifos, there is limited information regarding chlorpyrifos elimination by fungi (Singh et al. 2004; Li et al. 2007; Xu et al. 2008; Anwar et al. 2009). Fungi are responsible for the biogeochemical cycle and the degradation of hazardous materials in the biosphere (Liang et al. 2005). The first report concerning chlorpyrifos bioremediation using chlorpyrifos hydrolase produced in *C. cladosporioides* was performed by Gao et al. (2012).

## Conclusion and future prospects

Fungi are one of the most important groups of organisms on the planet, therefore, additional research to better characterise fungi should be performed. Recognised as an important and novel resource for natural bioactive products, fungi have potential applications in various domains such as medicine, the food industry and agriculture. Recently, research has been oriented towards investigating novel, natural bioactive products derived from fungi because of the declining discovery rate of active novel chemical entities. *C. cladosporioides* has been poorly studied despite its rich bioactive compound content, production of several enzymes and decreased risk compared with other extensively studied fungi. To date, there is scarce information regarding the bioactive compounds and secreted enzymes present in *C. cladosporioides*. However, studies that have been conducted on *C. cladosporioides* discovered bioactive compounds including p-methylbenzoic acid, EP and calphostin C as well as enzymes such as PME, PG and chlorpyrifos hydrolase.

p-Methylbenzoic acid stimulates the synthesis of 1,5-benzodiazepine and its derivatives that possess beneficial human health effects. It also increases PET yield, which is used in synthetic fibres, beverage, food and liquid containers. p-Methylbenzoic acid enhances EPA production, which is present in membrane-bound organisms from bacteria to humans. EPA may also potentially be used in the clinical management of malignancy patients. Although EP has demonstrated anticancer, antiangiogenic, antibacterial, anti-oxidative and immunosuppressive properties, *C. cladosporioides* derived EP has not been studied against various types of diseases. Calphostin C, a known anticancer agent, inhibits PKC by inactivating both PKC-epsilon and PKC-alpha. Calphostin C induces apoptosis in WEHI-231 cells and VSMCs. Calphostin C has been evaluated as a potential photodynamic therapy agent because of its stimulation of ER stress in certain cancers. Methylesterase (PME) and PG are pectinase enzymes that have garnered attention because of their applications in the food processing industry and its critical role in plant physiology. Chlorpyrifos hydrolase has been demonstrated to efficiently degrade and eliminate chlorpyrifos from the environment.

In conclusion, the focus of future research should be (1) investigating the optimal growth conditions for *C. cladosporioides* to produce high yields of p-methylbenzoic acid, EP and calphostin C along with chlorpyrifos hydrolase enzymes; (2) examining *C. cladosporioides* derived EP as a bioactive product against diverse types of diseases; (3) examining the anticancer activity of calphostin C in vivo; (4) characterising and completely purifying (PME) and PG; (5) characterising the molecular biology of the chlorpyrifos hydrolase gene and its regulatory mechanisms; and (6) identifying novel compounds and enzymes and examining those which exhibit biotechnological properties for use in medical and industrial applications.
